# Neuroprotective effects and mechanisms of action of nicotinamide mononucleotide (NMN) in a photoreceptor degenerative model of retinal detachment

**DOI:** 10.18632/aging.202453

**Published:** 2020-12-29

**Authors:** Xiaohong Chen, João A. Amorim, Giannis A. Moustafa, Jong-Jer Lee, Zhen Yu, Kenji Ishihara, Yasuhiro Iesato, Paulo Barbisan, Takashi Ueta, Konstantina A. Togka, Lin Lu, David A. Sinclair, Demetrios G. Vavvas

**Affiliations:** 1 Angiogenesis Laboratory, Massachusetts Eye and Ear, Harvard Medical School, Boston, MA 02114, USA; 2 State Key Laboratory of Ophthalmology, Zhongshan Ophthalmic Center, Sun Yat-sen University, Guangzhou 510060, China; 3 Department of Ophthalmology, Retina Service, Massachusetts Eye and Ear, Harvard Medical School, Boston, MA 02114, USA; 4 Department of Genetics, Blavatnik Institute, Paul F. Glenn Center for Biology of Aging Research, Harvard Medical School, Boston, MA 02115, USA

**Keywords:** nicotinamide mononucleotide, photoreceptor degeneration, NAD^+^, SIRT1, neuroprotection

## Abstract

Currently, no pharmacotherapy has been proven effective in treating photoreceptor degeneration in patients. Discovering readily available and safe neuroprotectants is therefore highly sought after. Here, we investigated nicotinamide mononucleotide (NMN), a precursor of nicotinamide adenine dinucleotide (NAD^+^), in a retinal detachment (RD) induced photoreceptor degeneration. NMN administration after RD resulted in a significant reduction of TUNEL^+^ photoreceptors, CD11b^+^ macrophages, and GFAP labeled glial activation; a normalization of protein carbonyl content (PCC), and a preservation of the outer nuclear layer (ONL) thickness. NMN administration significantly increased NAD^+^ levels, SIRT1 protein expression, and heme oxygenase-1 (HO-1) expression. Delayed NMN administration still exerted protective effects after RD. Mechanistic *in vitro* studies using 661W cells revealed a SIRT1/HO-1 signaling as a downstream effector of NMN-mediated protection under oxidative stress and LPS stimulation. In conclusion, NMN administration exerts neuroprotective effects on photoreceptors after RD and oxidative injury, suggesting a therapeutic avenue to treating photoreceptor degeneration.

## INTRODUCTION

Major photoreceptor degenerative diseases are primarily age-related eye disorders, leading to severe vision impairment or irreversible vision loss. These include age-related macular degeneration [1], diabetic retinopathy [2, 3] and retinal detachment (RD) [4, 5]. Despite distinct differences between these diseases, the separation of photoreceptors from the underlying retinal pigment epithelium (RPE) [4–7] or a loss of functional RPE [1, 8, 9], and eventual photoreceptor death is common to all of them. Since photoreceptors are highly metabolic [10], nutrient deprivation from the separated RPE induces the pathological responses that result in permanent neuronal loss. Fortunately, the separation of photoreceptors from the RPE (RD) can be well-modeled in animals [11–13]. By using the RD model, we and others have previously identified apoptosis [14, 15], regulated necrosis [13], oxidative stress [13, 16], and inflammation [17–19] as major pathophysiological changes after the separation. However, currently, no pharmacological approaches have been proven effective in treating photoreceptor degeneration in human clinical trials.

Nicotinamide adenine dinucleotide (NAD^+^) is a co-factor in redox metabolism that is critical to energy production [20]. In recent years, NAD^+^ decline has been identified in various diseases. Enhancing NAD^+^ biosynthesis is beneficial in age-associated metabolic and neurodegenerative disorders [21–23].

In recent years, several papers have reported NAD^+^ biosynthesis in eye diseases. Mutations in NMNAT1, an enzyme in the NAD^+^ salvage pathway, is found to cause Leber congenital amaurosis [24, 25]. Restoration of NAD^+^ appears to be important in vision, and that NMN has been reported to protect photoreceptors against light-induced retinal damage in rodent models [26, 23]. The NAD^+^-dependent silent information regulator 2 (Sir2) is among the major effectors for NAD^+^ beneficiaries and plays fundamental roles in aging, metabolism, cancer, stress response, neuronal function, inflammation, apoptosis and DNA repair [27–30]. SIRT1 has recently been found essential in retinal development and survival. Alteration of SIRT1 activity has been found in aged retina [30, 31], diabetic retinopathy [32, 33], light-induced retinal degeneration [34] and oxygen-induced ischemic retinopathy (OIR) [35], though much has to be done to validate these findings.

To date, however, little is known about the role of NAD^+^ when photoreceptors are separated from the RPE and whether the NAD^+^ and SIRT1 may play a role in protecting photoreceptors during their separation from the underlying RPE. In this study, we evaluated the effects of boosting NAD^+^ on the retina and photoreceptors after the induction of RD and explored the mechanisms of observed actions.

## RESULTS

### NMN supplementation reduces photoreceptor cell death in the early phase of RD

**Figure 1:**
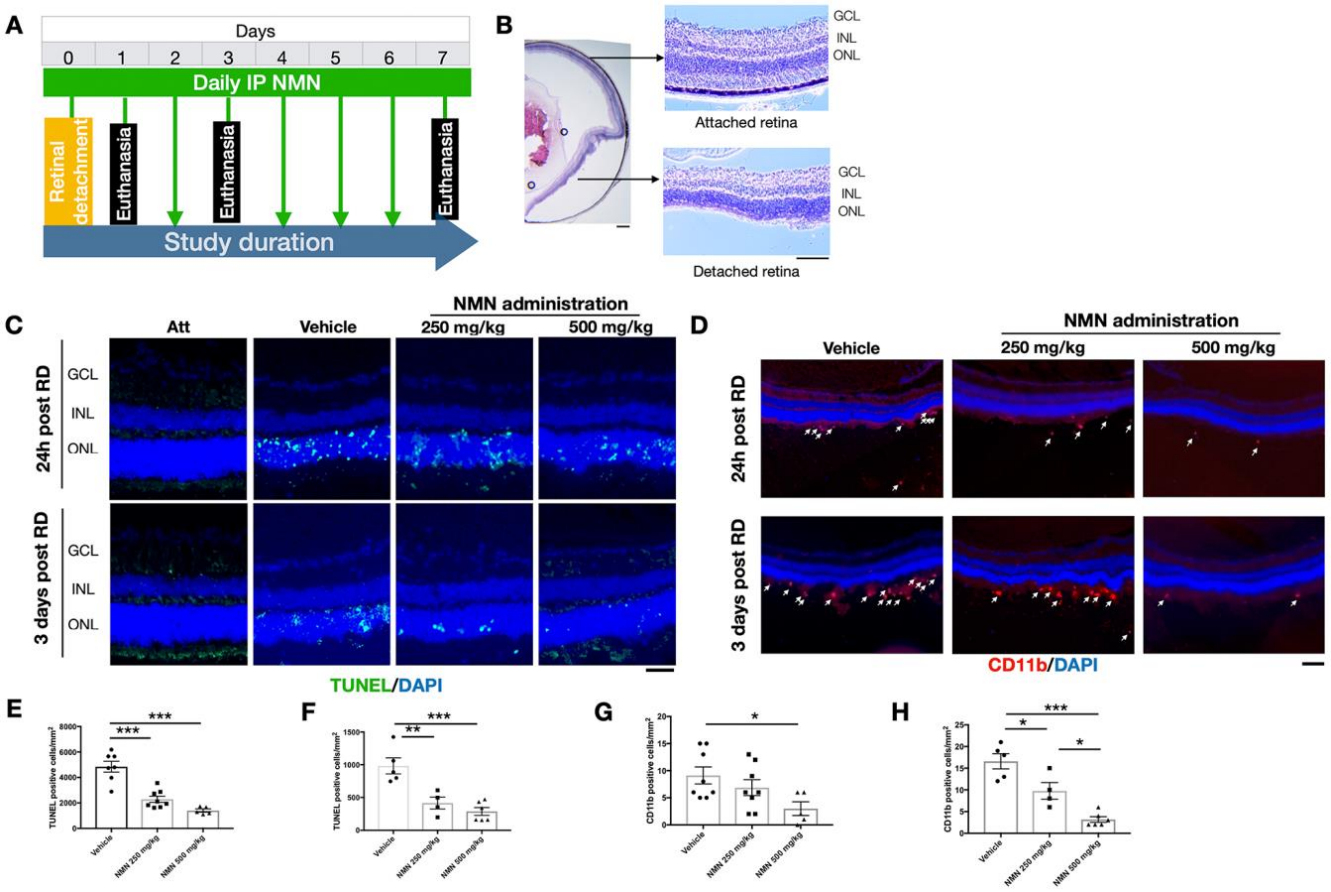
**Protective effects of NMN administration in the early phase of retinal detachment (RD).** (**A**) A flow chart for the *in vivo* experiments. (**B**) schematic anatomy of a mouse eye by hematoxylin and eosin (HE) staining showing a bullous after successful RD surgery. Scale bar: 50 μm. (**C**, **E**, **F**) TUNEL^+^ cells (green) were seen with the highest numbers in vehicle-treated retinas and lowest in the 500 mg/kg NMN-treated retinas at both 24h (**E**) and three days (**F**) post RD. Nuclei were counterstained with DAPI (blue). N = 4 to 8 eyes per group. Scale bar: 50 μm. (**D**, **G**, **H**) CD11b^+^ macrophages (red) infiltrated in the subretinal space after RD, NMN administration significantly reduced the number as soon as 24h post RD (**G**), and in a dose-dependent manner after three days of RD (**H**). Nuclei were counterstained with DAPI (blue). N = 5 to 8 eyes per group. Scale bar: 100μm. Statistical significance was analyzed with one-way ANOVA followed by Tukey-Kramer adjustments. *p<0.05, **p<0.01, ***p<0.001. Data are mean ± SEM.

The *in vivo* experimental procedures of RD were successfully induced in mouse eyes (Figure 1A, 1B), resulting in photoreceptor cell death which was attenuated after NMN administration at 24 hours after RD (Figure 1C, 1E, 1F). NMN at 250 mg/kg reduced cell death numbers by 52.7% (2292 ± 690 cells/mm^2^, *p<*0.001) whereas NMN at 500 mg/kg decreased cell death by 71.0% (1405 ± 290 cells/mm^2^, *p<*0.001).

Similar effects were seen 3 days after RD with NMN reducing the number of TUNEL^+^ cells by 57.8% in the 250 mg/kg NMN group (984 ± 274 cells/mm^2^ in vehicle vs. 415 ± 179 cells/mm^2^ in NMN; *p<*0.01) and 70.8% in the 500 mg/kg group (287 ± 147 cells/mm^2^; *p<*0.001).

These results show that daily supplementation of the NAD^+^ precursor NMN is effective in inhibiting cell death at the acute phase of photoreceptor injury, with the most protective results achieved at the dose of 500 mg/kg.

### NMN supplementation suppresses retinal inflammation

Our results suggested that 24 hours after RD induction, there was an accumulation of CD11b^+^ cells in the subretinal space in the vehicle RD group (9 ± 4 cell/mm^2^) (Figure 1D–1G). The number was reduced by an increasing dose of NMN from 250 mg/kg (7 ± 4 cell/mm^2^) to 500 mg/kg (3 ± 3 cells/mm^2^; *p<*0.05 compared to vehicle group).

The number of CD11b^+^ cells peaked three days post RD with the highest number in the vehicle group (17 ± 4 cells/mm^2^) followed by fewer cells in the 250 mg/kg NMN group (10 ± 4 cells/mm^2^; *p<*0.05 compared to vehicle group) and the lowest number in the 500 mg/kg NMN group (3 ± 2 cell/mm^2^, *p*< 0.001 compared to vehicle group) (Figure 1D–1H). Taken together, these results suggest that NMN supplementation is associated with suppressed inflammation after RD in a dose-dependent manner.

### NMN normalizes oxidative stress and upregulates antioxidant HO-1 after RD

**Figure 2:**
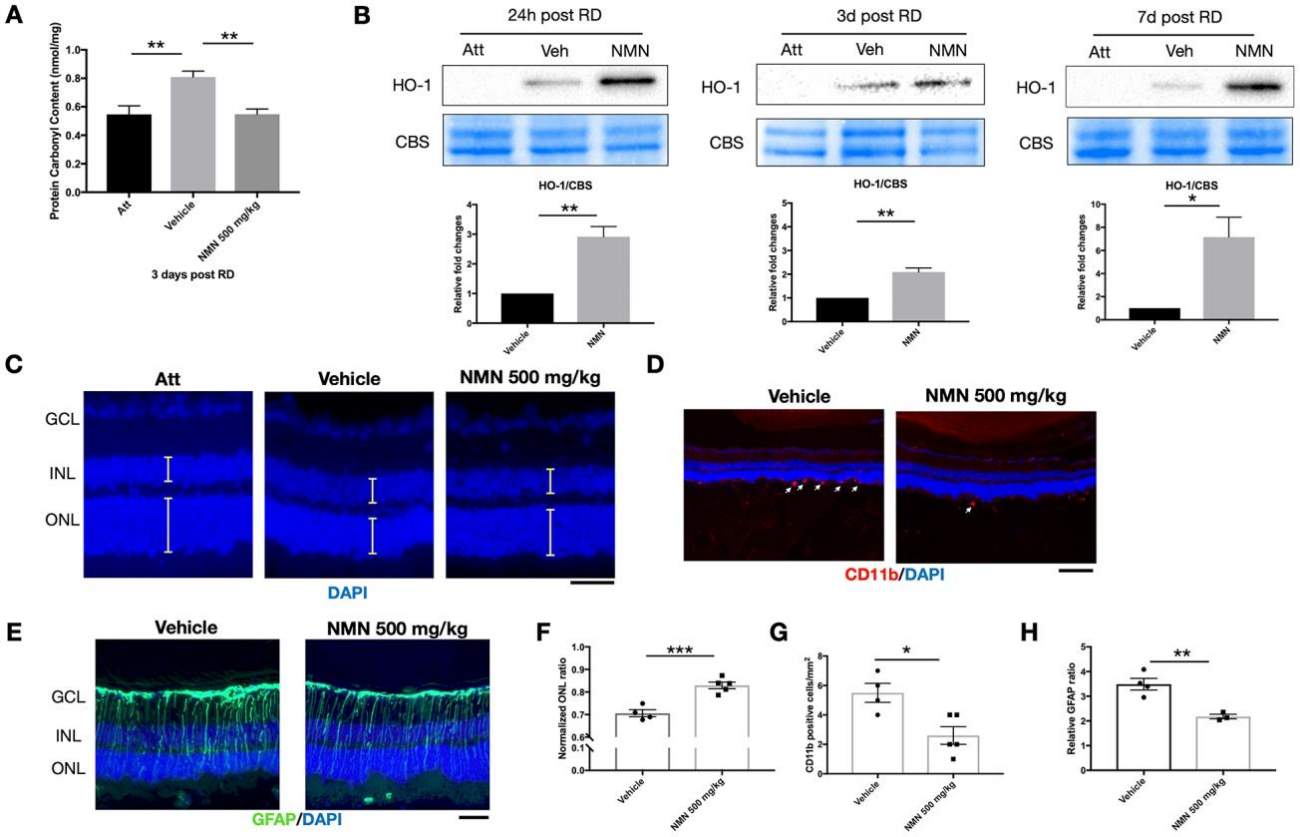
**NMN administration attenuates oxidative stress and is protective in the late phase of retinal detachment (RD).** (**A**) Protein carbonyl content (PCC) was significantly higher after RD while NMN treatment normalized PCC level comparable to the attached retinas at three days post RD. N = 6 eyes per group. (**B**) Heme oxygenase 1 (HO-1) was significantly up-regulated in the NMN groups as soon as 24h and remained highly expressed throughout the experimental period. N = 4 to 5 eyes per group. (**C**–**F**) Preservation of outer nuclear layer (ONL) thickness after NMN administration was seen 7 days after RD. N = 4 to 5 eyes per group. Scale bar=50 μm. The thickness of the layers was measured as the yellow lines indicated. (**D**–**G**) Inhibition of neuroinflammation seen by a reduced number of CD11b^+^ infiltrating macrophages (red) in NMN-treated retinas compared to vehicle-treated retinas. N = 4 to 5 eyes per group. Scale bar: 100 μm. (**E**–**H**) Reduced reactive gliosis represented by GFAP (green) staining in NMN-treated retinas compared to vehicle-treated retinas. N = 3 to 4 eyes per group. Scale bar: 50 μm. Statistical significance was analyzed with one-way ANOVA followed by Tukey-Kramer adjustments or the unpaired Student's t-test. *p<0.05. **p<0.01. ***p<0.001. Data are mean ± SEM.

We have previously identified elevated reactive oxygen species (ROS) as major pathophysiological changes after RD [13, 16]. We, therefore, investigated the effect of NMN supplementation on oxidative stress in the retina. Consistent with our previous results, there was a significant increase in oxidative stress as measured by protein carbonyl content (PCC) in the detached retinas (0.81 ± 0.1 nmol/mg) compared to the attached retinas (0.55 ± 0.15 nmol/mg; *p<*0.01) three days after RD (Figure 2A). NMN administration completely abolished this consequence (0.55 ± 0.1 nmol/mg, *p<*0.01 compared to vehicle) and maintained the tissue PCC comparable to the normal level as in the attached retina.

To elucidate the antioxidant effect of NMN after RD, we further found that HO-1, an inducible isoform of heme oxygenase, was significantly upregulated in the NMN-treated retinas (Figure 2B). HO-1 expression was barely detectable in the attached retinas. In contrast, under stress induced by RD, HO-1 expressions were detectable in protein level, and NMN administration highly upregulated HO-1 levels as soon as 24 hours and throughout the whole experimental period. Our results demonstrate that NMN administration can counteract excessive oxidative stress in the detached retina, and this effect is possibly executed through the upregulation of HO-1.

### NMN supplementation preserves ONL thickness and has an overall protection to the retina

To evaluate the long-term effect of NMN administration on photoreceptors after RD, we examined the overall impact on photoreceptor cell survival by measuring ONL thickness on Day 7 post RD. Administration of NMN at 500 mg/kg significantly preserved the retinal thickness seen by normalized ONL ratio (0.83) as compared to the vehicle-treated group (0.71, *p<*0.001) (Figure 2C–2F). Along with the increased survival of photoreceptors, NMN had a continued inhibitory effect of infiltrating CD11b^+^ macrophages (3 ± 1 cell/mm^2^) compared to the vehicle-treated retina (6 ± 1 cell/mm^2^, *p<*0.05) (Figure 2D–2G), and a significant suppression of gliosis as measured by GFAP immunofluorescence (*p<*0.01) (Figure 2E–2H). These results further demonstrated the beneficial effect of NAD^+^-boosting molecules on regulating retinal homeostasis after injury.

### NMN increases NAD^+^ levels and SIRT1 expression after RD

**Figure 3:**
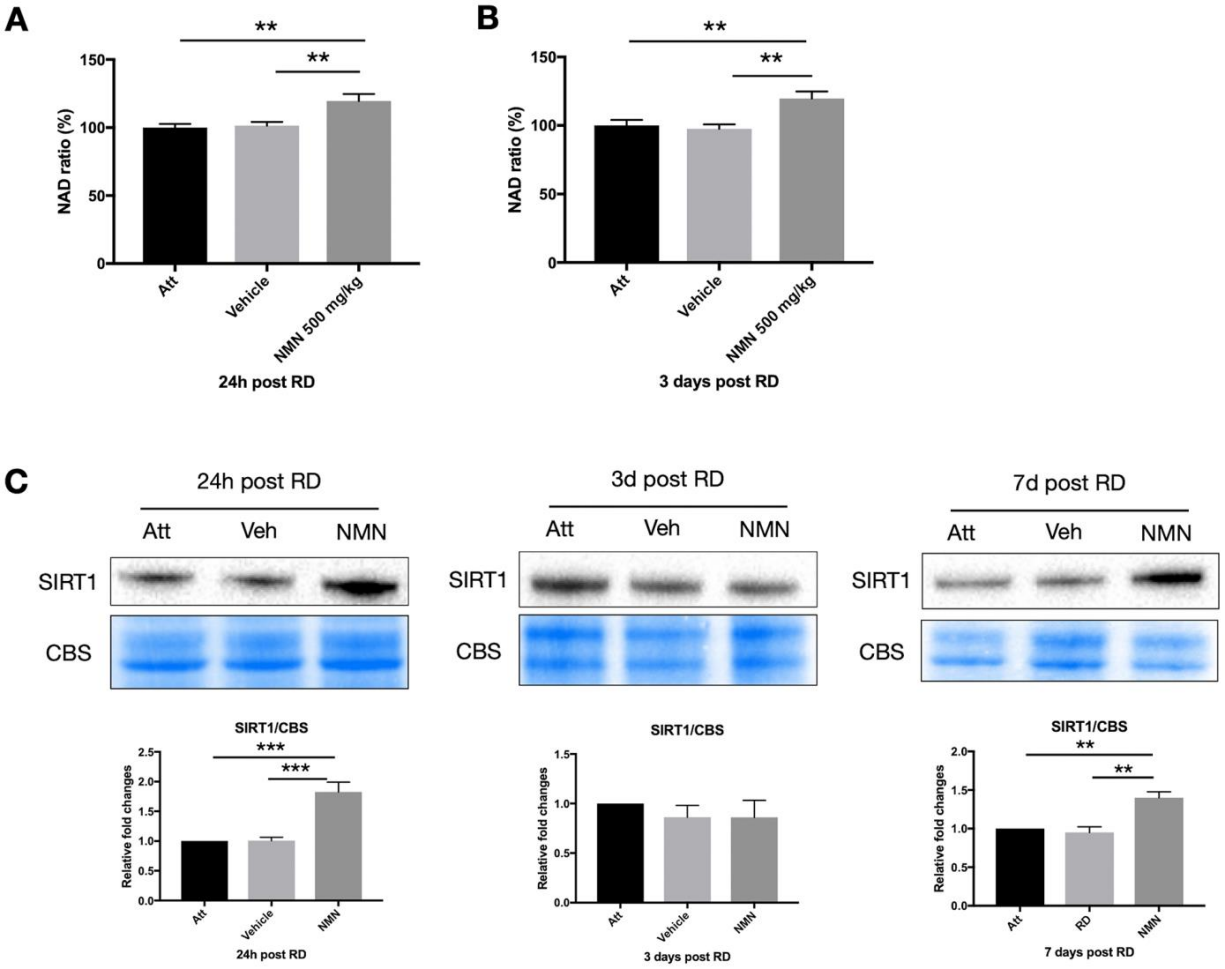
**Increased NAD^+^ and SIRT1 levels after retinal detachment (RD) with NMN supplementation.** Significant increase of NAD^+^ levels in the NMN-treated detached retinas compared to the vehicle and the attached retinas at 24h (**A**) and three days (**B**) after RD. NAD^+^ levels in vehicle-treated detached retinas were not lower compared to untreated controls. N = 8 to 11 eyes per group. (**C**) Upregulation of SIRT1 protein levels in NMN-treated retinas seen at 24h and 7 days post RD. SIRT1 levels were comparable among groups at three days post RD. N = 3 to 7 eyes per group. Statistical significance was analyzed with one-way ANOVA followed by Tukey-Kramer adjustments. *p<0.05. **p<0.01. ***p<0.001. Data are mean ± SEM.

NAD^+^ deficiency has been identified among the primary causes leading to many neurodegenerative diseases [20, 22, 23], and the NAD^+^ precursors have beneficial effects in protecting neurons by replenishing the NAD^+^ pool in the majority of studies. However, we did not observe any change of NAD^+^ level in the vehicle-treated detached retina at neither 24h (NAD ratio of vehicle group 101.5% vs. attached retina 100%, *p*>0.05) or three days (NAD ratio of vehicle group 97.5% vs. attached retina 100%, p>0.05) post RD compared to the attached normal retina (Figure 3A, 3B). Whereas NMN administration significantly increased the NAD^+^ above normal levels in both time points (24h: 119.5%, *p*=0.005 compare to attached; 3 days: 119.7%, *p*=0.008 compare to attached). Similar to the results from the retina, NMN administration significantly increased NAD^+^ levels compared to both vehicle and the attached RPE/choroid (Supplementary Figure 1).

To further elucidate the mechanism of NMN protection in the retina after increasing NAD^+^ levels, we examined the sirtuins, and found an upregulation of SIRT1 expression in the NMN-treated retinas at 24h and seven days after RD (Figure 3C). Retinal detachment alone did not induce the upregulation of SIRT1. This expression pattern was very similar to NAD^+^ content. SIRT1 expression did not differ significantly among groups three days after RD. Thus, SIRT1 may be necessary at different time points to confer protection after NMN administration, or they may have distinct and differing roles in photoreceptor survival.

### *In vitro* study replicated the protection of NMN and revealed an association to be in part with the SIRT1/HO-1 signaling

**Figure 4:**
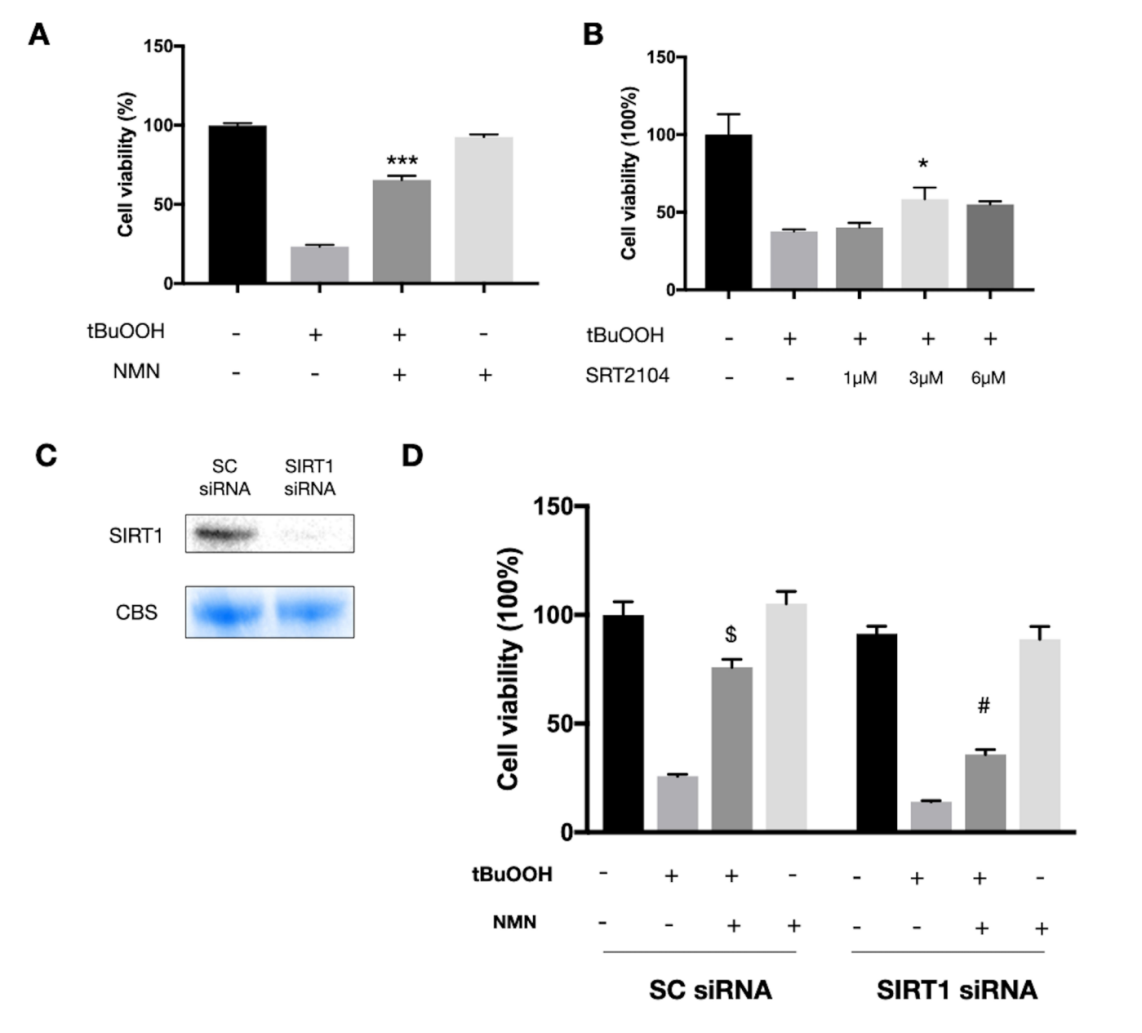
**Protected effects of NMN on 661W cells under oxidative stress are partially SIRT1 dependent.** (**A**) NMN administration significantly increased 661W cell viability after ROS insult. ***p<0.001 to tBuOOH only group. N = 4 to 5 per group. (**B**) SRT2104, a SIRT1 direct activator, had a similar effect as NMN in protecting 661W cells from ROS insult. *p<0.05 to tBuOOH only group. N = 3 to 4 per group. (**C**) SIRT1 was knocked down by siRNAs. (**D**) Silencing SIRT1 by siRNAs attenuated the protective effect of NMN after ROS insult compared to the scrambled (SC) siRNAs control group. $p<0.001 to tBuOOH only group in SC siRNA condition. #p<0.001 to tBuOOH and NMN treated group in SC siRNA condition. N = 4 to 8 per group. Statistical significance was analyzed with one-way ANOVA followed by Tukey-Kramer adjustments. Data are mean ± SEM.

Since we observed a very high oxidative stress status in the detached retina, we next tested the NMN mechanism of action *in vitro* using the photoreceptor-like cell line 661W in an oxidative stress model. A significant increase in cell survival was seen in the NMN administration group after ROS insult (Figure 4A). SRT2104, a direct SIRT1 activator, was also found protective to 661W cells after ROS insult (Figure 4B). However, the baseline level of NAD^+^ was also declined in the oxidative stress model (Supplementary Figure 2A). To examine if SIRT1 plays a role in mediating the effects of NMN supplementation, we transfected the 661W cells with SIRT1 siRNAs (Figure 4C). Our results showed a significantly lower level of cell survival in the SIRT1 siRNA transfection groups compared to the scramble siRNA control groups under NMN administration after ROS (Figure 4D). These results suggested that SIRT1 is at least partially responsible for the antioxidant property of NMN.

**Figure 5:**
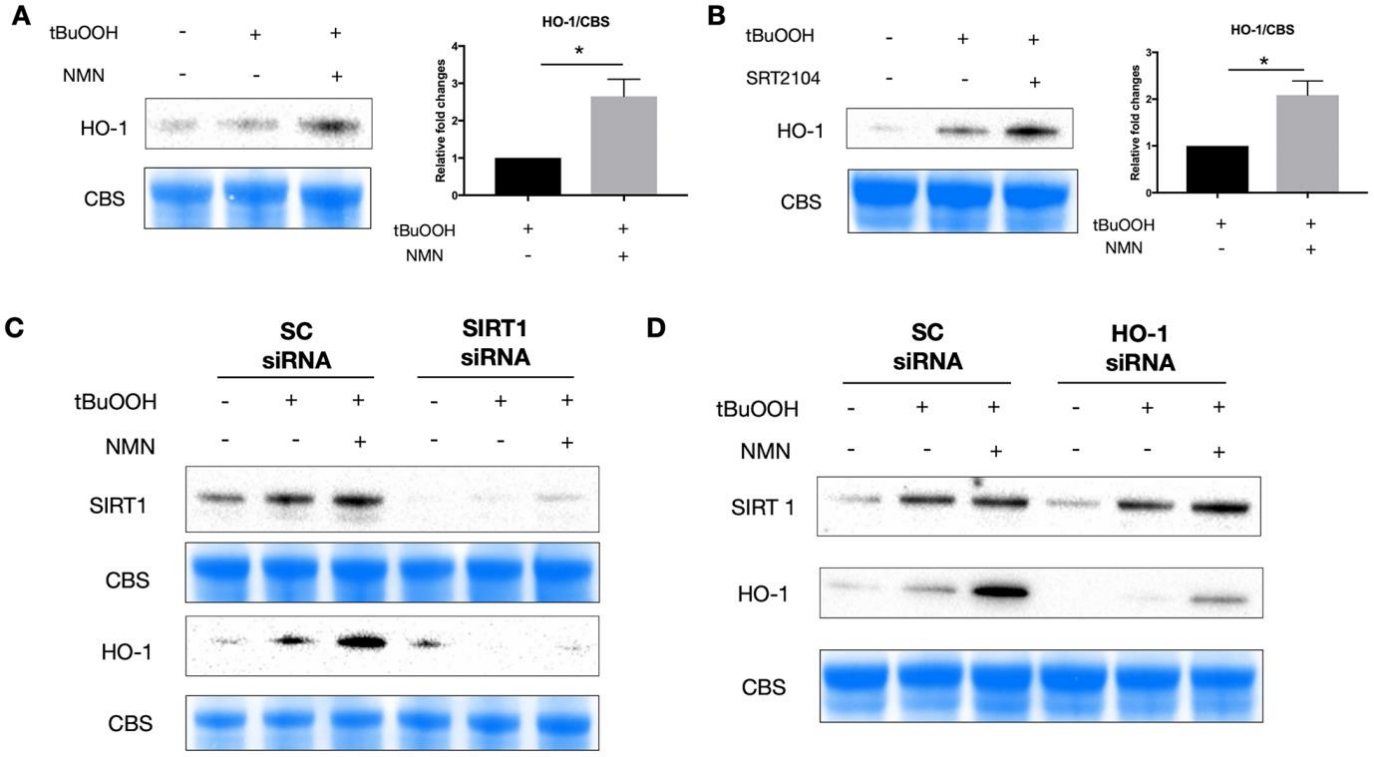
**A potential SIRT1/HO-1 signaling under the NMN-treated oxidative stress model.** Both NMN (**A**) and SRT2104 (**B**) administration significantly increased HO-1 protein levels after ROS insult. N = 4 per group. Statistical significance was analyzed with the unpaired Student's t-test. *p<0.05. **p<0.01. ***p<0.001. Data are mean ± SEM. (**C**) Silencing SIRT1 by siRNA almost abolished the induced HO-1 expression by NMN after ROS insult. (**D**) HO-1 siRNA didn’t alter the expression of SIRT1 by NMN after ROS insult.

Using western blot analysis, we found an upregulation of HO-1 protein expression after both NMN (Figure 5A) and SRT2104 treatment (Figure 5B) in the oxidative stress model. Knocking down of SIRT1 almost totally inhibited the induction of HO-1 with NMN administration (Figure 5C), whereas knocking down HO-1 didn’t alter the expression of SIRT1 under the NMN treated conditions (Figure 5D) suggesting that HO-1 upregulation after NMN was the downstream of SIRT1 upregulation.

### NMN and SIRT1 exerted protective effects directly on 661W cells not through action on immune cells in an *in vitro* model of immune mediated toxicity to photoreceptor cells

**Figure 6:**
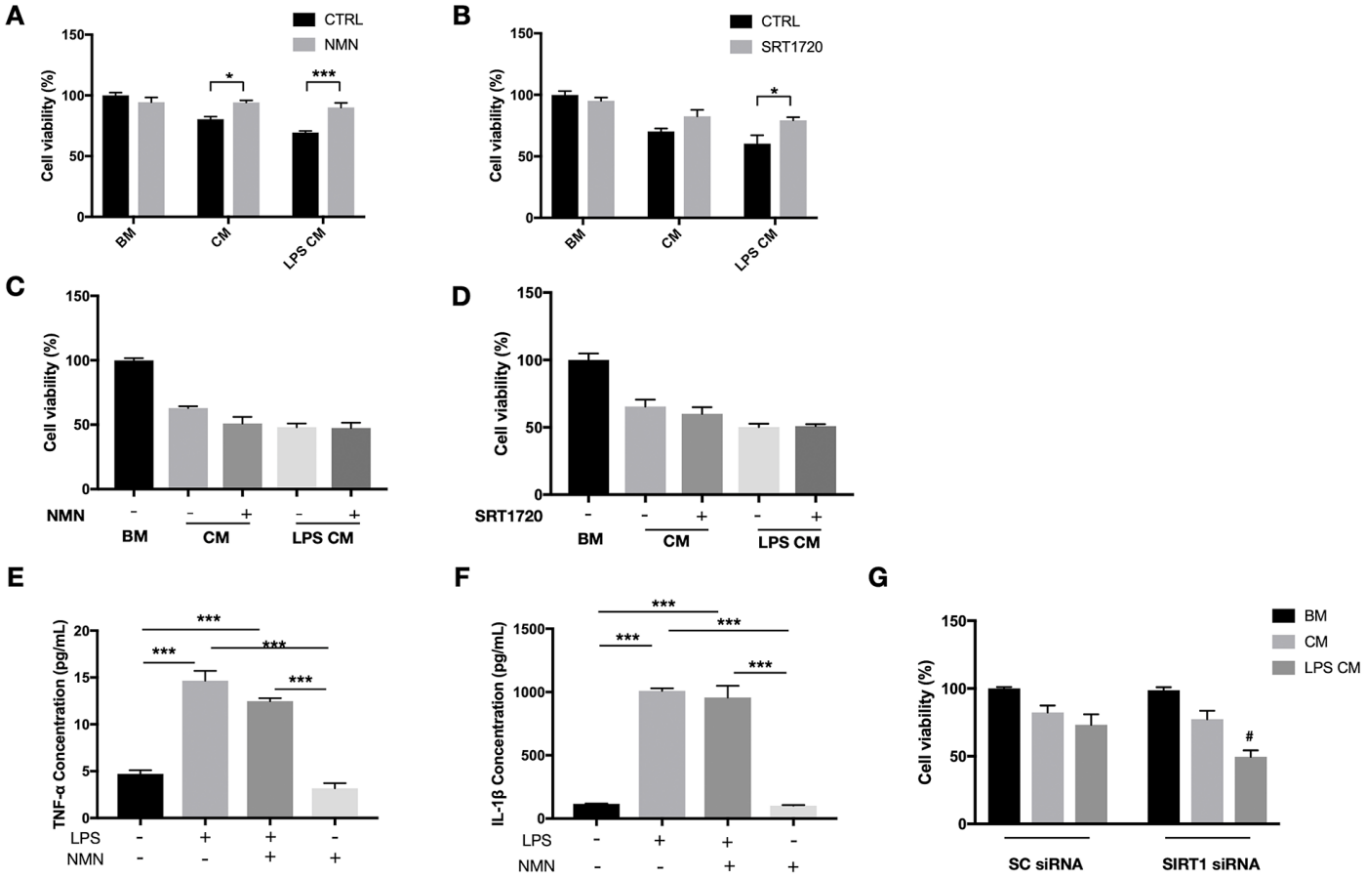
**Protective effects of NMN and SIRT1 activation to 661W cells in RAW264.7 conditioned media (CM).** (**A**) NMN administration significantly increased the cell viability of 661W cells in both the LPS treated and untreated RAW264.7 CM. N = 3 to 4 per group. (**B**) SRT1720 also showed a protective effect to 661W cells in the LPS treated RAW264.7 CM. N = 4 per group. The CM from neither NMN (**C**) or SRT1720 (**D**) treated RAW264.7 cells had protective effects to 661W cells. N = 4 to 6 per group. LPS can induce significant increases in both TNF-α (**E**) and IL-1β (**F**) expressions, which were not altered by NMN administration. N = 3 per group. (**G**) SIRT1 siRNA in 661W cells significantly reduced cell viability in the LPS stimulated RAW264.7 CM. #p<0.05 to LPS CM treated group in SC siRNA condition. N = 3 to 4 per group. Statistical significance was analyzed with the unpaired Student's t-test between each treatment and its control, or with one-way ANOVA followed by Tukey-Kramer adjustments. *p<0.05. **p<0.01. ***p<0.001. Data are mean ± SEM.

Our *in vivo* results have shown a significant reduction of infiltrating macrophages in the subretinal space after the NMN supplement. To determine whether this result is a direct anti-inflammatory effect of NMN or indirectly from the reduced photoreceptor cell death, we examined the role of NMN and SIRT1 in a RAW264.7 macrophage conditioned media (CM) toxicity model. CM samples from RAW264.7 cells treated or without the LPS were both toxic to 661W cells. NMN administration significantly increased the viability of 661W cells in both the LPS treated and untreated RAW264.7 CM (Figure 6A). The direct SIRT1 activator SRT1720 also showed a protective effect on 661W cells in the LPS treated RAW264.7 CM (Figure 6B).

Next, we administrated NMN or SRT1720 directly to RAW264.7 cells (stimulated with or without LPS), and subsequently, CM was collected and used to treat 661W cells. Neither NMN- nor SRT1720-treated RAW264.7 cells resulted in any protective effects on 661W cells, in contrast to those 661W cells that were directly treated with either molecules (Figure 6C, 6D). These results indicated that the protective effects of NMN and SRT2104 were probably acting directly on 661W cells, rather than by altering the toxicity of RAW264.7 CM. In order to see whether NMN administration has effect on the inflammatory cytokines, we treated RAW264.7 cells with LPS, in the presence or absence of NMN. The CM samples were collected, and the expression of neurotoxic cytokines TNF-α and IL-1β were assessed by ELISA. These results showed that LPS can induce significant increases in both TNF-α (Figure 6E) and IL-1β (Figure 6F). However, NMN administration at 1 mM didn’t significantly alter the levels of both cytokines.

Lastly, we used SIRT1 siRNAs to knockdown SIRT1 in 661W cells before treating them with RAW264.7 CM with or without LPS. Knocking down SIRT1 subjected 661W cells to a higher sensitivity to the neurotoxic effects of LPS stimulated CM from RAW264.7 cells, as can be seen by the significantly lower cell viability compared to the control group (Figure 6G).

### Delayed NMN supplementation continues to exert protection to the retina after RD

**Figure 7:**
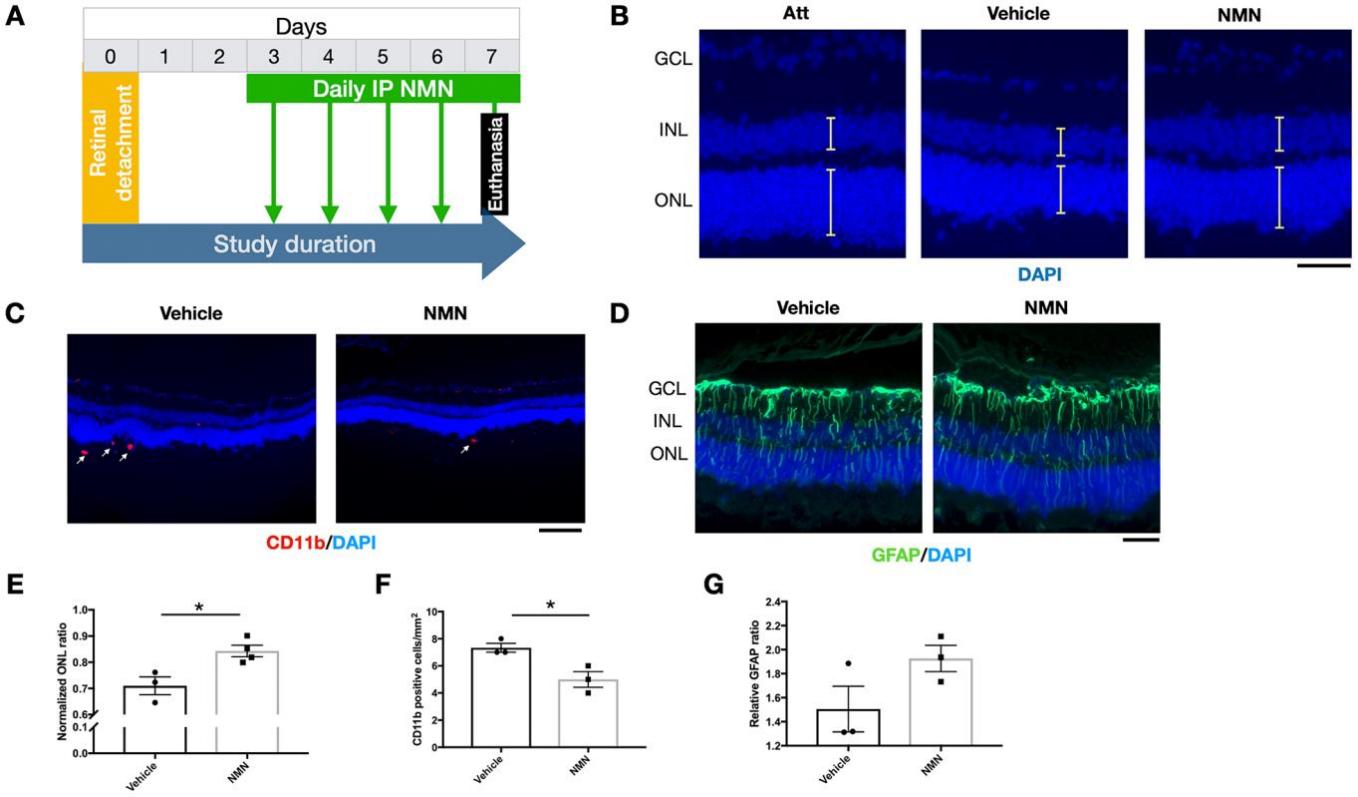
**Protective effects of delayed NMN administration to retinal detachment (RD).** (**A**) A flow chart for the *in vivo* experiments. (**B**–**E**) Delayed NMN administration still have protective effect to outer nuclear layer (ONL) thickness 7 days post RD. N = 3 to 4 eyes per group. Scale bar=50 μm. (**C**–**F**) Reduced number of CD11b^+^ infiltrating macrophages (red) in delayed NMN-treated groups compared to vehicle. N = 3 eyes per group. Scale bar: 100 μm. (**D**–**G**) No significance was found between NMN and vehicle treated retinas of the GFAP (green). N = 3 eyes per group. Scale bar: 50 μm. Statistical significance was analyzed with the unpaired Student's t-test. *p<0.05. **p<0.01. ***p<0.001. Data are mean ± SEM.

To be more relevant to clinical settings, we administrated NMN at Day 3 post RD and evaluated the effects on the retina at Day 7 post RD (Figure 7A). Our results showed a preserved ONL thickness in comparison to the vehicle group (Figure 7B–7E), along with an inhibitory effect of infiltrating CD11b^+^ macrophages compared to the vehicle group (Figure 7C–7F). GFAP was unaltered (Figure 7D–7G). These results showed that delayed NMN administration can still exert protective effect after RD.

## DISCUSSION

Our study provides evidence of neuroprotective effects of NMN, a NAD^+^-boosting molecule, in photoreceptor degeneration after RD. NMN administration can attenuate neuroinflammation by reducing CD11b^+^ infiltrating macrophages, decreasing oxidative stress, upregulating HO-1 expression, and inhibiting photoreceptor cell death by reducing TUNEL^+^ cells, thus achieving an overall protection of the retina. Although RD was not associated with NAD^+^ deficiency, NMN administration increased retinal NAD^+^ levels, upregulated SIRT1, and HO-1 at various time points. *In vitro* studies revealed a neuroprotective effect of NMN in ROS-induced 661W cell death probably through the SIRT1/HO-1 signaling. Moreover, NMN and SIRT1 protected 661W cells cultured in the RAW264.7 CM by a direct protection to the cells rather than altering the toxicity of the inflammatory CM. Lastly, delayed NMN administration continues to show protection to the retina after RD. Taken together, our results suggest a potential therapeutic value of NMN administration in treating photoreceptor degeneration in clinical settings.

We have shown previously that photoreceptor separation from underlying RPE triggers ROS production [13, 16]. Here, we show that NMN treatment *in vivo* normalized excessive ROS production and increased the antioxidant responses. In particular, increasing NAD^+^ level above normal was associated with a significant upregulation of HO-1 protein level. HO-1 (also named HMOX1 or HSP32) is a stress-inducible heat shock protein that catalyzes heme into biliverdin, carbon monoxide, and iron, exerting anti-oxidative and anti-inflammatory functions [36–40]. HO-1 is induced in the detached retina but is insufficient to completely counteract the damage of RD. The higher level of HO-1 induced by NMN enabled neuroprotective outcomes. Several other studies have also suggested activation of antioxidant response after NAD^+^ precursors [41–43], with one of the studies showing that increased recruitment of Nrf2 to HO-1 gene promoter after NMN supplement [43].

Contrary to most studies showing a decline of NAD^+^ levels in neurodegenerative models, our results did not show NAD^+^ levels to be significantly altered after RD. However, the NAD^+^-boosting molecule NMN was associated with neuroprotective effects with the NAD^+^ levels above physiologic. Nonetheless, if this supraphysiologic elevation of NAD^+^ is a mere association or partially causative remains unknown. Our finding is similar to a recent paper reporting the toxicity of SOD1 loss in an astrocyte cell line was not associated with reduced NAD^+^ levels or sirtuins, yet NMN administration increased survival and depended on sirtuin presence [43]. It is possible that young (7-8 week) adult mice used in our study may have the capacity to restore NAD^+^ level after RD, whereas older animals used in other neurodegenerative models may have limited capacity for NAD^+^ restoration under stress. Besides, NAD^+^ levels of 661W cells under oxidative stress was indeed declined (Supplementary Figure 2A) and the SIRT1 protein level increased (Supplementary Figure 2B). The differences in the observations can be attributed to the fact that the complex tissue of the retina responses to noxious stimuli differ from cell models. It may be that compensatory mechanism in cells other than photoreceptors counteract a potential NAD depletion in photoreceptors. Based on our results, we think that although NAD^+^ levels do not decline after experimental RD, NMN administration boosts NAD^+^ levels beyond physiologic, and this may help activate enzymes, such as SIRT1, that may play a protective role in the process.

SIRT1 has recently been found essential in retinal development and various pathological conditions [30–35, 44, 45]. Notably, we found that silencing SIRT1 didn’t completely abolish the protective effect of NAD^+^. Here are several possible reasons: 1) other sirtuin family members may also participate in the neuroprotection after the increase of NAD^+^. For example, both SIRT1 and SIRT2 can mediate neural stem and/or progenitor cell fate decisions into oligodendrocytes [46]. SIRT3 was shown to protect against mitochondrial fragmentation and neuronal cell death induced by SOD1 G93A overexpression [47]. SIRT6 is shown to extend lifespan by modulating telomeric chromatin [48, 49]. SIRT7 increases the stress resistance of cardiomyocytes and prevents apoptosis and inflammatory cardiomyopathy in mice [50]. 2) Antioxidant defenses may be regulated by other sirtuins. Accumulating studies revealed a complex relationship between sirtuins and ARE defense systems, with several sirtuins (SIRT1, 2, 3, 5 and 6) participating in the regulation of various antioxidant genes in multiple cells, tissues and disease models [42, 51–53]. Besides, a disruption of redox homeostasis is also capable of affecting the expression level, post-translation, and interactions of sirtuins [36]. 3) NAD^+^ metabolism alone is sufficient to mediate various biological processes independent of sirtuins [54]. For example, changes in the NAD^+^/NADH ratio affect the production of superoxide by complex I [55–57], and directly influenced ROS and the antioxidant defenses. Thus, it is critical to determine whether the protective effects via boosting NAD^+^ levels are mediated via changes to the NAD^+^/NADH ratio directly or via activation of sirtuins [54–58].

We observed a significant reduction of infiltrating macrophages in the subretinal space after the NMN supplement *in vivo*. The subsequent *in vitro* outcome revealed a direct protective effect to 661W cells instead of indirectly altering the toxicity of RAW264.7 CM by NMN or SIRT1 activator. The NAD^+^ biosynthesis pathway and SIRT1 are important regulators in the immune system. NAD^+^ and SIRT1 have been reported to suppress inflammation in various diseases such as in autoimmune encephalomyelitis (EAE) [59], liver ischemia-reperfusion injury [39], high-fat-diet obesity [60], etc. It is reported that SIRT1 can reduce pro-inflammatory responses by deacetylating the p65 subunit of nuclear factor kappa B (NF-κB) [61] The NAD biosynthesis pathways regulates the macrophages and act as the metabolic switch that determine the immune responses under specific settings [62, 63].

The limitations of our study that can also serve as future directions are the following: 1) although we have shown a benefit of NMN administration in the acute phase of RD, the long-term effect and safety of NMN administration in the retina of mice and humans needs to be evaluated. A study of one-year oral administration of NMN showed safe and well-tolerated effects in wild-type C57BL/6 mice, and human clinical studies have been performed with no apparent negative effects [64]; 2) more work is needed to elucidate the exact molecular role of SIRT1 and identify its substrate in regulating the antioxidant pathways and mitochondrial homeostasis; 3) other sirtuin family members, as well as other NAD^+^-dependent enzymes, such as PARPs, CD38 may also play a role in the retinal homeostasis, which requires further investigation. 4) the investigation of the pharmacokinetics of NMN and the metabolism of NAD^+^ in photoreceptor cells will be fundamental to understanding retinal neuroprotection and how to dose the molecule in humans. 5) a further elucidation of the anti-inflammatory effects of NMN and SIRT1 in the retina after RD is needed.

In conclusion, our study demonstrates the neuroprotective effects of NMN supplementation in rescuing photoreceptor degeneration after RD. NMN treatment was associated with an increase in NAD^+^ and SIRT1 levels in the injured retina, leading to anti-apoptotic, anti-inflammatory, and antioxidant effects. The therapeutic effect of NMN may occur at least partially through the activation of SIRT1/HO-1 signaling. These results provide an impetus for studies in larger animals and humans with photoreceptor degeneration in a clinical setting.

## MATERIALS AND METHODS

### Animals

Wildtype C57BL/6 mice (7-10 weeks old) were purchased (Jackson Laboratory, Bar Harbor, ME, USA) and fed *ad libitum* on standard laboratory chow and water in an air-controlled room with a 12-h light/12-h dark cycle. All animal experiments adhered to the Association for Research in Vision and Ophthalmology (ARVO) Statement for the Use of Animals in Ophthalmic and Vision Research under the approval by the Animal Care Committee of Massachusetts Eye and Ear Infirmary.

### Induction of experimental retinal detachment

The induction of our rodent retinal detachment model has been addressed in detail in our previously published paper from our lab [64]. We used this model to achieve reproducible and sustainable bullous of RD. Briefly, after general anesthesia, pupils were dilated and proparacaine eye drops (Sandoz Inc., Princeton, NJ, USA) were applied. The conjunctiva was opened, and a self-sealing scleral tunnel was created followed by a corneal paracentesis to lower intraocular pressure. Next, a 34-gauge needle connected to a 10μl syringe (Hamilton Company, Reno, NV, USA) was inserted through the scleral tunnel and 2 μl of 1% sodium hyaluronate (Provisc; Alcon, Fort Worth, TX, USA) were injected to separate the neurosensory retina from RPE. The scleral wound was sealed by Webglue (Patterson Companies, Mendota Heights, MN, USA). Antibiotic ointment (Bacitracin Zinc Ointment; Fougera Pharmaceuticals Inc, Melville, NY, USA) was applied followed by analgesia. Mice were kept on a heating pad with careful monitoring until fully awake.

### NMN administration and experimental groups

We administrated NMN (Bontac Bio-engineering (Shenzhen) CO.,LTD) at doses of 250 mg/kg/d and 500 mg/kg/d by diluting in sterile PBS for our study based on the effective dose in previous studies ranges from 62.5 mg/kg to 500 mg/kg by intraperitoneal injections [23, 65–67].

Mice were divided into the following groups: normal attached control group (Att), RD with PBS injection group (Vehicle), RD with 250 mg/kg NMN group and RD with 500 mg/kg NMN group. On the day of surgery, mice were undergoing the induction of RD followed by NMN or vehicle injections half an hour later. Daily injections of NMN or vehicle were then performed at the end of the light phase. Retina tissues were harvested at 3 time points: 24 hours, 3 days and 7 days post RD. A separate group of mice received NMN administration at Day 3 post RD, and then with daily injections of NMN till the Day 7 post RD.

### Hematoxylin and eosin (HE) staining

Sections were prefixed in 4% paraformaldehyde (PFA) and then stained with Gill’s I hematoxylin (Sigma) and eosin (Sigma).

### TUNEL assay and normalized ONL thickness ratio

A *TdT-dUTP terminal nick-end labeling* (TUNEL) assay was performed following the manufacturer’s protocol (ApopTag Fluorescein *In Situ* Apoptosis Detection Kit; MilliporeSigma, Burlington, MA, USA). Pictures were taken with an upright AXIO Imager.M2 Zeiss fluorescence microscope under a 10×/0.3 lens (Zeiss EC-PLAN NEOFLUAR, Carl Zeiss Inc., Thornwood, NY, USA). TUNEL positive cells were quantified by using the Image J software (developed by Wayne Rasband, National Institutes of Health, Bethesda, MD, USA). Any samples with hemorrhage, cataract, or shrunk part of the retina were excluded.

The thickness ratio of the ONL and corresponding inner nuclear layer (INL) can be measured in DAPI stained slides in the detached and attached part of the retina by masked observers. The ‘‘normalized ONL thickness ratio’’ is defined as the (ONL thickness/neuroretina thickness in the detached retina)/(ONL thickness/neuroretina thickness in the attached retina) as previously described [13].

### Immunofluorescence

Sections were fixed in 4% paraformaldehyde (PFA), blocked with 0.5% bovine serum albumin (BSA) with 0.3% Triton X-100 in PBS, and incubated overnight at 4 ° C with anti-CD11b antibody (BD Pharmingen, BD Biosciences, San Jose, CA, USA) and anti-GFAP antibody (Proteintech, Rosemont, IL, USA). The secondary antibodies used in the study were Alexa-Fluor 647 goat anti-rat antibody and Alexa-Fluor 488 goat anti-rabbit antibody (Molecular Probes, Thermo Fisher Scientific, Waltham, MA, USA). Finally, sections were counterstained with DAPI (AnaSpec/Eurogentec Group) and mounted with Fluoromount-G (SouthernBiotech, Birmingham, AL, USA).

The total number of CD11b^+^ cells in the subretinal space was counted, and the area of subretinal space was measured. The results were expressed as: CD11b^+^ cells (cells/mm^2^) = total cell number/area of subretinal space.

The glial fibrillary acidic protein (GFAP) is a sign of the reactive gliosis including the astrocytes and Müller cells. [68, 69]. We measured the area of GFAP and the total area from ILM to OLM in each section. The results were expressed as: GFAP ratio = (GFAP area /total area of ILM to OLM in the detached retina)/(GFAP area /total area of ILM to OLM in the attached retina).

### NAD^+^ level measurement

NAD^+^ levels were determined following the instructions of a commercial NAD/NADH Quantification Kit (Sigma, St. Louis, MO, USA).

### Protein carbonyl content (PCC)

The amount of PCC in the retina was measured by using a protein carbonyl ELISA kit (Oxiselect; Cell Biolabs, San Diego, CA) according to the manufacturer’s instructions.

### Cell culture and treatment

The photoreceptor-derived cell line 661W was kindly provided by Dr Miayyad Al-Ubaidi (University of Houston, TX, USA) [70] and was maintained at 37° C under a humidified atmosphere of 5% CO_2_.

The oxidative stress model was induced by tert-Butyl hydroperoxide (tBuOOH, ACROS Organics, Geel, Belgium). The cell viability tests were performed in 10 mM tBuOOH to evaluate the following conditions: 1 mM NMN administration, SRT2104 administration, or SIRT1 siRNA transfection with NMN administration. Protein expressions in Figure 5 were examined by western blots after treatment with 5 mM tBuOOH.

In the macrophage CM toxicity model, RAW264.7 cells were cultured and stimulated with or without LPS. The CM samples were collected to culture 661W. Cell viability tests were performed to determine the effects of NMN or SRT1720 administration. Besides, NMN or SRT1720 was also administrated in the RAW264.7 cells with or without LPS stimulation, CM was collected to culture 661W, and the cell viability was tested under these conditions.

### Gene silencing by siRNA transfection

Transfection of siRNAs was performed by mixing Lipofectamine RNAiMAX Reagent (Thermo Fisher Scientific, Waltham, MA USA) with SIRT1 siRNAs according to the manufacturer’s instruction. A scramble siRNA (sc siRNA) was used separately as a negative control. All siRNAs were purchased from Origene (Rockville, MD, USA).

### Cell viability test

We used the Cell Counting Kit (CCK-8, Dojindo, Rockville, Maryland, USA) to perform the cell viability tests according to the manufacturer’s instructions. Briefly, after the treatment, the medium was replaced with fresh culture medium containing 10% (v/v) CCK-8 reagent. The absorption at 450 nm was measured with multiple times using a SpectraMax 190 microplate spectrophotometer (Molecular Device, San Jose, CA). Each experiment has been replicated for three or more times.

### Enzyme-linked immunosorbent assay (ELISA)

The expression levels of TNF-α and IL-1β were measured with Mouse TNF-α (R&D Systems, Inc., Minneapolis, MN) and mouse IL-1β/IL-1F2 (R&D Systems, Inc.) ELISA kits, according to the manufacturer’s protocol.

### Western blot analysis

After euthanasia, eyes were enucleated and retinas were extracted and homogenized in ice-cold mammalian protein extraction reagent (T-PER, Thermo Fisher, Waltham, MA) with protease/phosphatase inhibitor cocktail (Cell Signaling, Danvers, MA). Protein concentration was measured using a Pierce Coomassie (Bradford) protein assay. Western blots were performed with incubation of primary antibodies: HO-1 and SIRT1 from Proteintech followed by secondary antibodies. The results were visualized with HRP substrate reagent (Genetex, Irvine, CA) and detected with an ECL imaging system ChemiDoc MP (Bio-Rad Laboratories, Hercules, CA).

### Statistical analysis

Results are expressed as mean ± SEM. Statistical analysis between two groups was performed using an unpaired Student’s *t*-test. Multiple groups were analyzed by ANOVA followed by Turkey-Kramer adjustments. The significance levels were defined as P < 0.05 (*), P < 0.01 (**) and P < 0.001 (***). Graphs were plotted with Prism GraphPad Prism 7 (La Jolla, CA, USA).

## Supplementary Material

Supplementary Figures
