# Supplementary material for: Neuroprotective effects and mechanisms of action of nicotinamide mononucleotide (NMN) in a photoreceptor degenerative model of retinal detachment
Source: Aging (Albany NY). 2020 Dec 29;12(24):24504–21. doi: 10.18632/aging.202453 (PMC7803565; doi:10.18632/aging.202453)
Supplement: Supplementary Figures [file aging-12-202453-s001.pdf]

SUPPLEMENTARY FIGURES

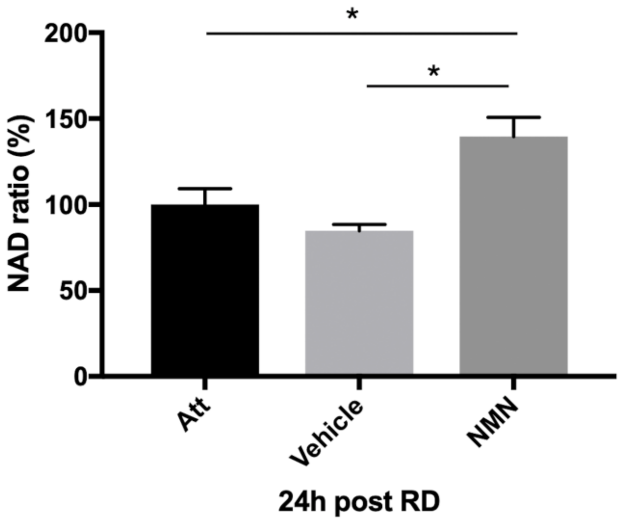

Supplementary Figure 1. NMN administration upregulates NAD⁺ levels in RPE/choroid complex units after retinal detachment (RD). A tendency of decreasing NAD⁺ levels was seen in vehicle-treated RPE/choroid compared to the attached, but no significance was found. N = 3 to 4 eyes per group. Statistical significance was analyzed with one-way ANOVA followed by Tukey-Kramer adjustments. *p<0.05. **p<0.01. ***p<0.001. Data are mean ± SEM.

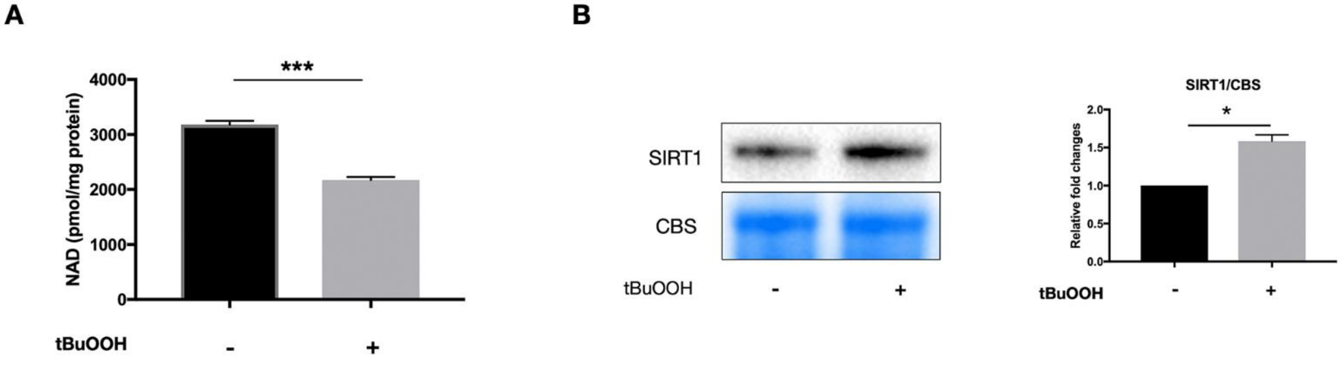

Supplementary Figure 2. Baseline NAD⁺ and SIRT1 level of 661W cells under oxidative stress. (**A**) NAD⁺ levels were significantly reduced in the oxidative stress model compared to control. N = 6 per group. (**B**) SIRT1 protein expressions were unregulated in the oxidative stress model compared to control. N = 3 per group. Statistical significance was analyzed with the unpaired Student's t-test. *p<0.05. **p<0.01. ***p<0.001. Data are mean ± SEM.
